# Identification of pyroptosis-associated genes with diagnostic value in calcific aortic valve disease

**DOI:** 10.3389/fcvm.2024.1340199

**Published:** 2024-01-25

**Authors:** Chenxi Yu, Yifeng Zhang, Ling Yang, Mirenuer Aikebaier, Shuyao Shan, Qing Zha, Ke Yang

**Affiliations:** ^1^ Department of Cardiovascular Medicine, Ruijin Hospital, Shanghai Jiaotong University School of Medicine, Shanghai, China; ^2^ Department of Cardiology, Shanghai Ninth People’s Hospital, Shanghai Jiaotong University School of Medicine, Shanghai, China

**Keywords:** calcific aortic valve disease, pyroptosis, machine learning, immune infiltration, GEO

## Abstract

**Background:**

Calcific aortic valve disease (CAVD) is one of the most prevalent valvular diseases and is the second most common cause for cardiac surgery. However, the mechanism of CAVD remains unclear. This study aimed to investigate the role of pyroptosis-related genes in CAVD by performing comprehensive bioinformatics analysis.

**Methods:**

Three microarray datasets (GSE51472, GSE12644 and GSE83453) and one RNA sequencing dataset (GSE153555) were obtained from the Gene Expression Omnibus (GEO) database. Pyroptosis-related differentially expressed genes (DEGs) were identified between the calcified and the normal valve samples. LASSO regression and random forest (RF) machine learning analyses were performed to identify pyroptosis-related DEGs with diagnostic value. A diagnostic model was constructed with the diagnostic candidate pyroptosis-related DEGs. Receiver operating characteristic (ROC) curve analysis was performed to estimate the diagnostic performances of the diagnostic model and the individual diagnostic candidate genes in the training and validation cohorts. CIBERSORT analysis was performed to estimate the differences in the infiltration of the immune cell types. Pearson correlation analysis was used to investigate associations between the diagnostic biomarkers and the immune cell types. Immunohistochemistry was used to validate protein concentration.

**Results:**

We identified 805 DEGs, including 319 down-regulated genes and 486 up-regulated genes. These DEGs were mainly enriched in pathways related to the inflammatory responses. Subsequently, we identified 17 pyroptosis-related DEGs by comparing the 805 DEGs with the 223 pyroptosis-related genes. LASSO regression and RF algorithm analyses identified three CAVD diagnostic candidate genes (TREM1, TNFRSF11B, and PGF), which were significantly upregulated in the CAVD tissue samples. A diagnostic model was constructed with these 3 diagnostic candidate genes. The diagnostic model and the 3 diagnostic candidate genes showed good diagnostic performances with AUC values >0.75 in both the training and the validation cohorts based on the ROC curve analyses. CIBERSORT analyses demonstrated positive correlation between the proportion of M0 macrophages in the valve tissues and the expression levels of TREM1, TNFRSF11B, and PGF.

**Conclusion:**

Three pyroptosis-related genes (TREM1, TNFRSF11B and PGF) were identified as diagnostic biomarkers for CAVD. These pyroptosis genes and the pro-inflammatory microenvironment in the calcified valve tissues are potential therapeutic targets for alleviating CAVD.

## 1 Introduction

Calcific aortic valve disease (CAVD) is the most common valvular disease in developed countries and is characterized by aortic valve fibrosis and calcification with a peak aortic flow velocity of >2 m/s (1). Therefore, CAVD is a serious public health burden with high mortality rates. The 4-year event-free survival rate is 16% for patients with asymptomatic and severe CAVD (2). The average survival period is 2–3 years for symptomatic patients without medical intervention (2). The prognosis of CAVD patients has improved significantly in the recent years with the development of surgical aortic valve replacement (SAVR) and transcatheter aortic valve implantation (TAVI) (3, 4). However, pathogenesis of CAVD is still unclear and effective therapeutics are currently not available to prevent or delay the development of CAVD (5). Therefore, there is an urgent need to identify effective biomarkers that would help guide clinical management and treatment of CAVD.

Pyroptosis is an inflammatory form of programmed cell death characterized by plasma membrane pore formation that leads to the release of cellular contents, including pro-inflammatory cytokines such as interleukin (IL)-1β and IL-18 (6). This process involves activation of the cysteinyl aspartate-specific proteinase (caspase)-1-induced canonical inflammatory pathway and the caspase-11-induced non-canonical inflammatory pathway (6). The canonical pathway is mediated by inflammasomes, formed by three components, PYCARD, also known as ASC, pyrin-containing receptors such as NLRP3 and pro-caspase-1 (7). Non-canonical pathway is triggered by the cytosolic lipopolysaccharides (6). Caspase-1 and caspase-11 were then activated and cleave gasdermin D (GSDMD) into N-GSDMD, an executor of pyroptosis (8). Recent studies have shown that pyroptosis is associated with cardiovascular diseases. Wei et al. and Díaz-García et al. reported that the NLRP3 inflammasome cascade promoted atherosclerosis development by enhancing pyroptosis (9, 10). Shi et al. reported that the caspase-11-induced cardiomyocyte pyroptosis played a significant role in myocardial I/R injury (11). Zeng et al. reported that NLRP3 inflammasome-mediated pyroptosis contributed to dilated cardiomyopathy (12). However, very few studies have investigated the role of pyroptosis in the occurrence and development of CAVD.

Therefore, in this study, we investigated the role of pyroptosis-related genes in CAVD and constructed a diagnostic model based on the candidate biomarker genes. We also analyzed the correlation between these pyroptosis-related diagnostic biomarkers and the immune cell types in the calcified valve tissues to determine the changes in the immune microenvironment in CAVD.

## 2 Methods and materials

### 2.1 Data sources and clinical samples

Four gene expression datasets were obtained from the Gene Expression Omnibus (GEO) database (https://www.ncbi.nlm.nih.gov/geo/). Among these, two microarray datasets, GSE51472 and GSE12644, included 30 samples (15 normal aortic valves and 15 calcific aortic valves). Log2 transformation was performed on the GSE51472 dataset. These two datasets from the GPL570 platform were combined using the “SVA” package of R language and used as the training dataset (13). The third microarray dataset, GSE83453, included 27 samples (8 normal aortic valves and 19 calcific aortic valves) and an RNA sequencing dataset, GSE153555, included 20 samples (10 normal aortic valves and 10 calcific aortic valves). The RNA sequencing dataset was filtered to remove low expression genes (at least 75% samples with gene count >0) and then normalized as counts per million (CPM) values using the “edgeR” package. These two datasets were used as the validation set. Furthermore, we downloaded 223 pyroptosis-related genes from the NCBI database.

Calcific aortic valves were obtained from patients with CAVD undergoing surgical treatment. Non-calcific aortic valves were acquired from cardiac transplant recipients. All aortic valves were obtained from Ruijin Hospital, Shanghai Jiao Tong University School of Medicine (Shanghai, China). The study protocol was approved by the Ethics Committee of Ruijin Hospital, Shanghai Jiao Tong University School of Medicine, and written informed consent was acquired from all patients.

### 2.2 Identification of differentially expressed genes

We performed principal component analysis (PCA) to determine the differential gene expression between the 30 samples. Differentially expressed genes (DEGs) were screened using the “LIMMA” package with p value <0.05 and |log2FC| >0.585 as threshold parameters. DEGs were visualized by generating a volcano plot and a heatmap plot using the “ggplot2” and “pheatmap” packages, respectively. Pyroptosis-related DEGs were identified by overlapping the DEGs and the pyroptosis-related genes using the Venn diagram.

### 2.3 Enrichment analysis

Functional enrichment analysis was performed to determine the enriched Gene Ontology (GO) terms and Kyoto Encyclopedia of Genes and Genomes (KEGG) pathways using the “clusterProfiler” package. GO analyses of the DEGs, the pyroptosis-related DEGs and the three diagnostic candidate genes were performed to determine the enriched biological processes (BP), cellular components (CC), and molecular functions (MF). KEGG pathway analyses were performed to identify the enriched signaling pathways represented by the DEGs and the pyroptosis-related DEGs. The enrichment analyses results were visualized using a bubble graph and a circle plot. The *P* value was adjusted by the Benjamini‒Hochberg method and the adjusted *p* value <0.05 was set as the cutoff value.

### 2.4 Machine learning and the diagnostic model construction

After identifying the pyroptosis-related DEGs, least absolute shrinkage and selection operator (LASSO) regression analysis and random forest (RF) machine learning algorithm were used to further filter the candidate genes and select the variables for CAVD. LASSO regression is a useful tool for variable selection and outcome prediction. RF is also a commonly-used machine learning algorithm to build multiple decision trees and generate a more accurate prediction. LASSO regression analysis was performed using the “glmnet” package. The lambda.min and lambda.1se were calculated and any lambda value between them was regarded reasonable. The lambda.1se value was used to select smallest number of genes in the appropriate range. RF analysis was performed using the “randomForest” package. The significance of genes was estimate using the Gini method by calculating the sum of the squares of residual errors. Top 5 genes with importance scores above 0.6 were selected (14). Then, the candidate diagnostic genes were identified by overlapping the candidate genes from the LASSO regression analysis and the RF analysis. The GSE153555 and GSE83453 datasets were then used to validate the expression levels of the candidate diagnostic genes. The diagnostic model was constructed by the RF method. The calibration plot and the decision curve analysis (DCA) of the model were generated using the “runway” and “dca” packages. Receiver operating characteristic (ROC) analysis was performed to evaluate the diagnostic efficacy of the model for predicting CAVD. The area under the curve (AUC) values >0.75 were considered as good performances. A user-friendly website was constructed to visualize the model using the “shiny” package.

### 2.5 Protein–protein interactions between diagnostic genes, pyroptosis-related genes and CAVD-related genes

To explore the role diagnostic genes in CAVD pathogenesis and the underlying signaling networks involved in pyroptosis and CAVD, 3 diagnostic genes, 5 pyroptosis-related genes and 3 CAVD-related genes were selected based on previous research (15, 16). Pearson's correlation analysis was performed to illustrate the association between these three categories of genes. A protein-protein interaction (PPI) network was constructed using the STRING database (https://string-db.org). Cytoscape software (Version 3.10.0) was applied to visualize the PPI network. Red dots represent upregulated genes, and blue dots represent downregulated genes.

### 2.6 Immune infiltration analysis

Previous studies have reported that CAVD and pyroptosis are associated with immune responses (7, 17). CIBERSORT is an analytical method to estimate the immune cell composition using tissue gene expression data (18). We analyzed the status of immune cell infiltration and the proportions of 22 types of immune cells in the normal and CAVD tissues of the training group and validation groups using the “CIBERSORT” package (https://cibersort.stanford.edu/index.php). The RNA sequencing dataset, GSE153555 was normalized as transcripts per kilobase million (TPM) values using the “IOBR” package according to the requirement of CIBERSORT (19). Bar plots were applied to present the proportion of diverse immune cells in each sample. Box plots were generated to compare the percentages of immune cells between normal and CAVD groups. Pearson's correlation analysis was performed to determine the association between proportions of different immune cell types and the expression levels of the candidate diagnostic genes. The results were visualized using the “ggplot2” package.

### 2.7 Immunohistochemistry

Immunohistochemistry for valve samples was conducted as previously described (20). The collected calcific and non-calcific valves were fixed overnight in 4% paraformaldehyde and then sliced into frozen sections (5 μm thickness). The sections were then stained using Alizarin Red S for histological examination. To detect the concentration of caspase-1, sections were incubated with rabbit polyclonal antibodies against caspase-1 (1:200, ab74279, Abcam, MA, USA) and then incubated with horseradish peroxidase (HRP)-conjugated secondary antibodies (1:500, #8114, CST, MA, USA). All section images were photographed with a microscope (Olympus Microsystems).

### 2.8 Statistical analysis

The continuous variables between two groups were compared using the student's *t* test or the Kruskal–Wallis test. Pearson correlation analysis was performed to determine the association between immune cell types and the candidate diagnostic genes. *P* < 0.05 was considered statistically significant. Statistical analysis was performed using the R software version 4.2.2.

## 3 Results

### 3.1 Identification and enrichment analysis of DEGs in the calcified valves

**Figure 1:**
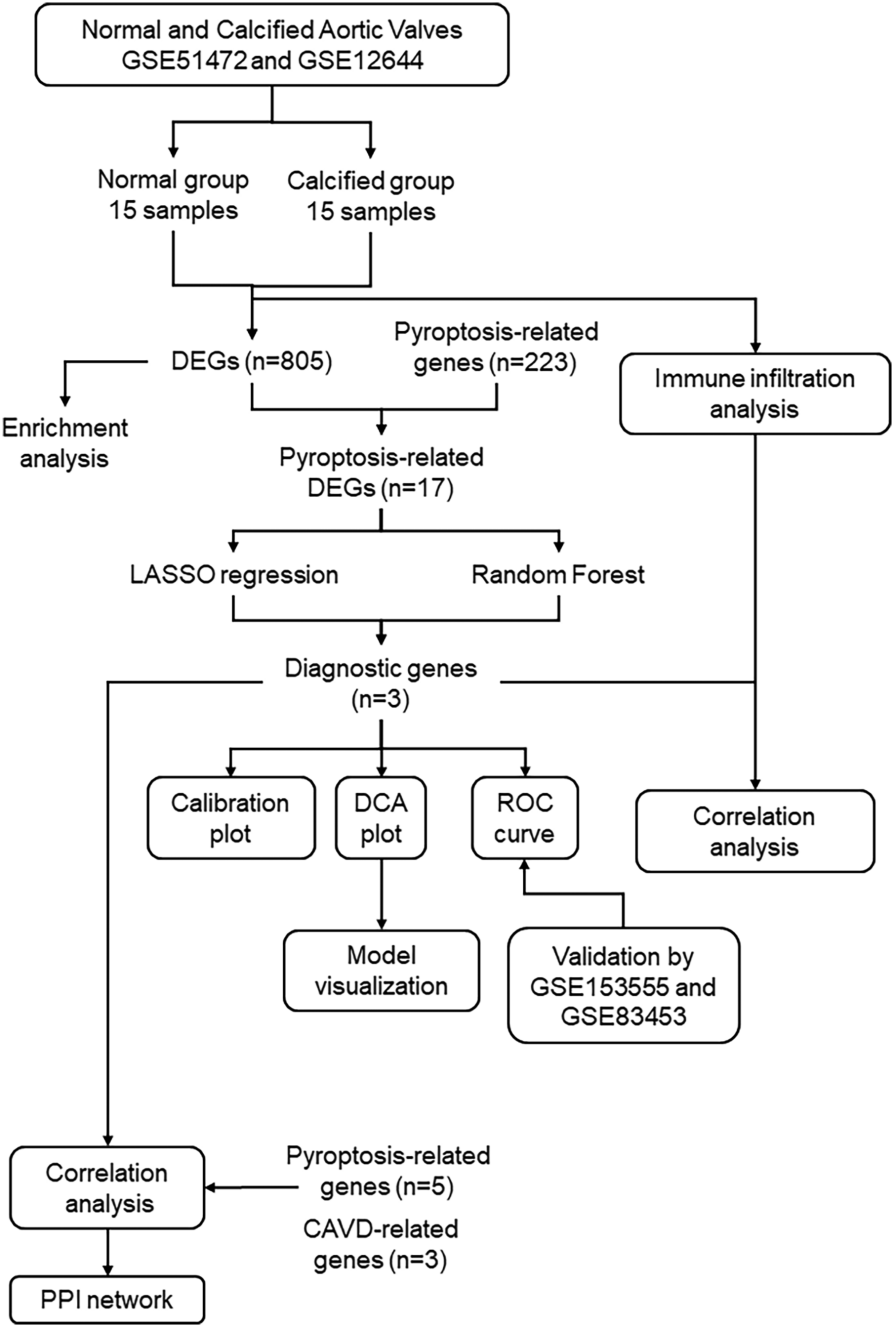
Study flowchart. GSE, gene expression omnibus series; DEGs, differentially expressed genes; LASSO, least absolute shrinkage and selection operator.

**Figure 2:**
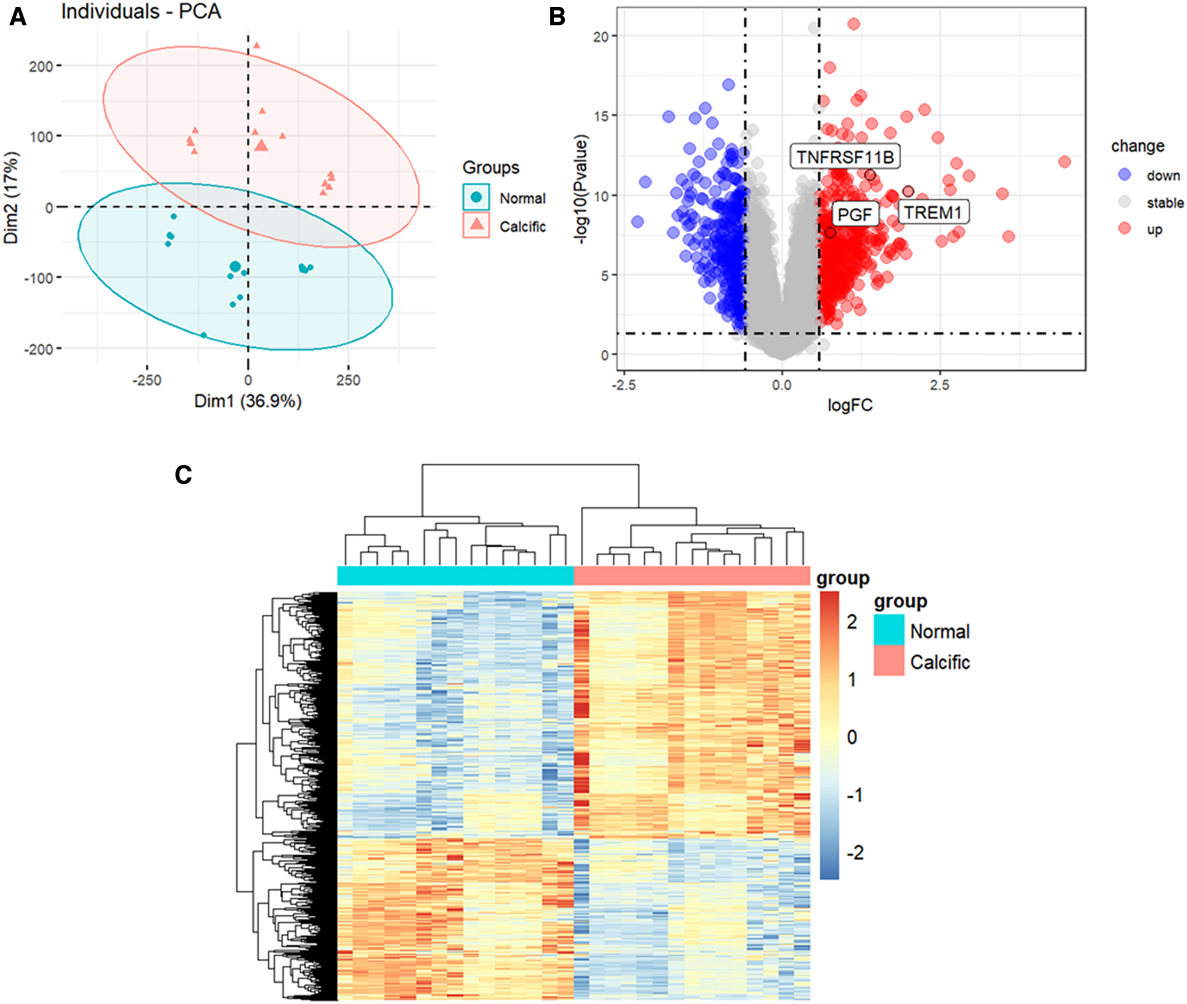
Identification of DEGs between normal and calcified aortic valves. (**A**) PCA for normal and calcified aortic valves. (**B**) Volcanic map of the DEGs between normal and calcified aortic valves based on *P* < 0.05 and |log2FC| >0.585 as threshold parameters. Red dots represent upregulated genes. Blue dots represent downregulated genes. (**C**) Heatmap of the DEGs. Upregulated genes are shown in red. Downregulated genes are shown in blue.

The study flowchart is shown in Figure 1. The gene expression profiles before and after batch correction for the combined samples (*n* = 30) from the GSE51472 and GSE12644 datasets are shown in Supplementary Figure S1. PCA was performed to identify the variations between the normal and calcified valvular samples. The first two components of PCA together accounted for 53.9% of the variations between the normal and calcified valvular samples, with 36.9% variations in the first principal component (PC1 or Dim1) and 17% variations in the second principal component (PC2 or Dim2) (Figure 2A). Differential gene expression analysis resulted in the identification of 805 DEGs, including 319 down-regulated genes and 486 up-regulated genes in the calcified valvular samples compared with the normal valvular samples (Figure 2B). The expression patterns of the DEGs in all the samples are shown in the heatmap (Figure 2C). We selected *TREM1*, *TNFRSF11B*, and *PGF* as potential diagnostic genes for the follow-up analysis. These 3 genes were up-regulated in the calcified valvular samples of the training cohort.

**Figure 3:**
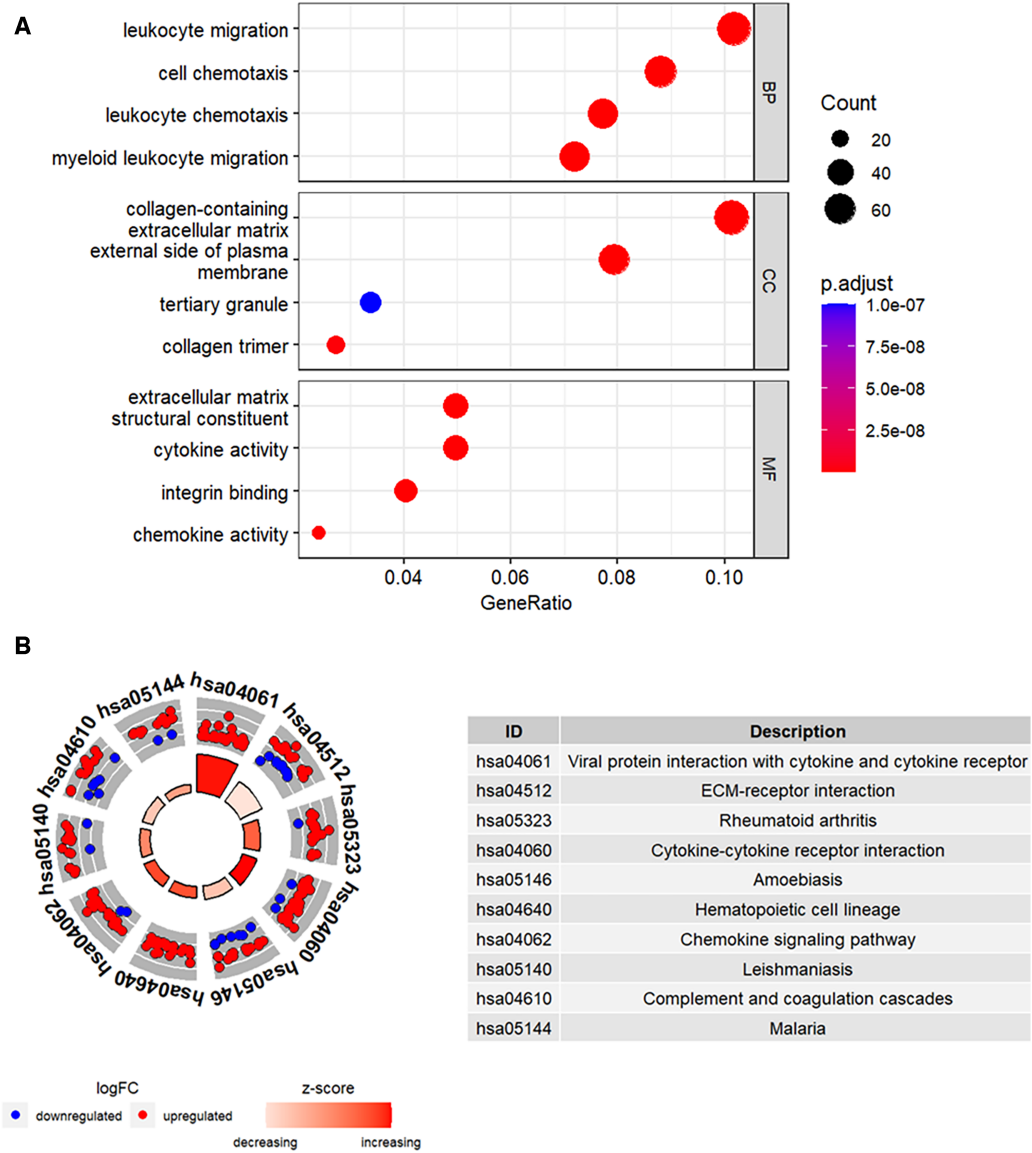
Functional enrichment analysis of the DEGs. (**A**) The top four GO terms (BP, CC, and MF) represented by the DEGs. (**B**) KEGG pathway analysis results of the DEGs. The top 10 KEGG pathways were identified based on the adjusted *P* values. The size of the sectors in the circle plot represents the adjusted *p* value of KEGG pathways.

Functional enrichment analysis was performed to further determine the biological functions of the DEGs. The results of GO enrichment analysis, including the top 4 enriched terms for the biological process (BP), cellular component (CC), and molecular function (MF) categories are shown in Figure 3A. DEGs were enriched in leukocyte migration and chemotaxis among the BP categories, collagen-containing extracellular matrix and external side of plasma membrane among the CC categories, and cytokine activity and chemokine activity among the MF categories. Figure 3B shows the top 10 enriched KEGG pathways, including cytokine-cytokine receptor interaction and chemokine signaling pathway.

### 3.2 Identification of pyroptosis-related DEGs between normal and calcified valvular groups

**Figure 4:**
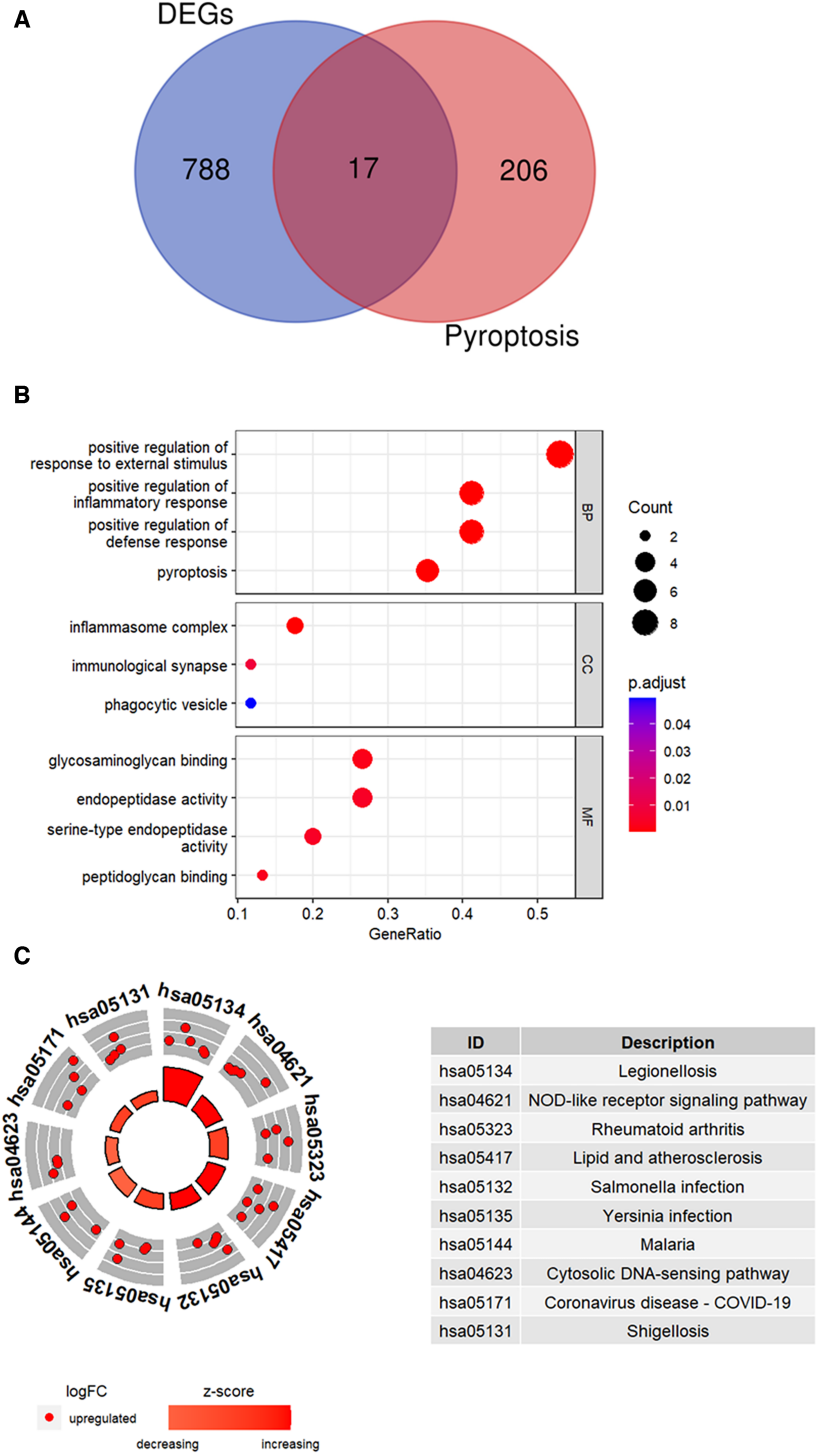
Identification of pyroptosis-related DEGs. (**A)** Venn plot shows overlapping genes between DEGs and pyroptosis-related genes. (**B**) GO analysis results for the pyroptosis-related DEGs. (C) The top 10 KEGG pathways of pyroptosis-related DEGs were identified based on the adjusted *P* values. The size of the sectors in the circle plot represents the adjusted *p* value of KEGG pathways.

DEGs (*n* = 805) and the pyroptosis-related genes (*n* = 223) were compared using a Venn plot and 17 pyroptosis-related DEGs were identified (Figure 4A). Functional enrichment analysis was performed to determine the biological functions of these pyroptosis-related DEGs. GO enrichment analysis is shown in Figure 4B. The pyroptosis-related DEGs were enriched in the positive regulation of inflammatory response and pyroptosis among the BP categories, inflammasome complex and immunological synapse among the CC categories, and glycosaminoglycan binding and endopeptidase activity among the MF categories. Figure 4C shows the top 10 enriched KEGG pathways of pyroptosis-related DEGs, including NOD-like receptor signaling pathway and lipid and atherosclerosis.

### 3.3 Identification of diagnostic genes for CAVD

**Figure 5:**
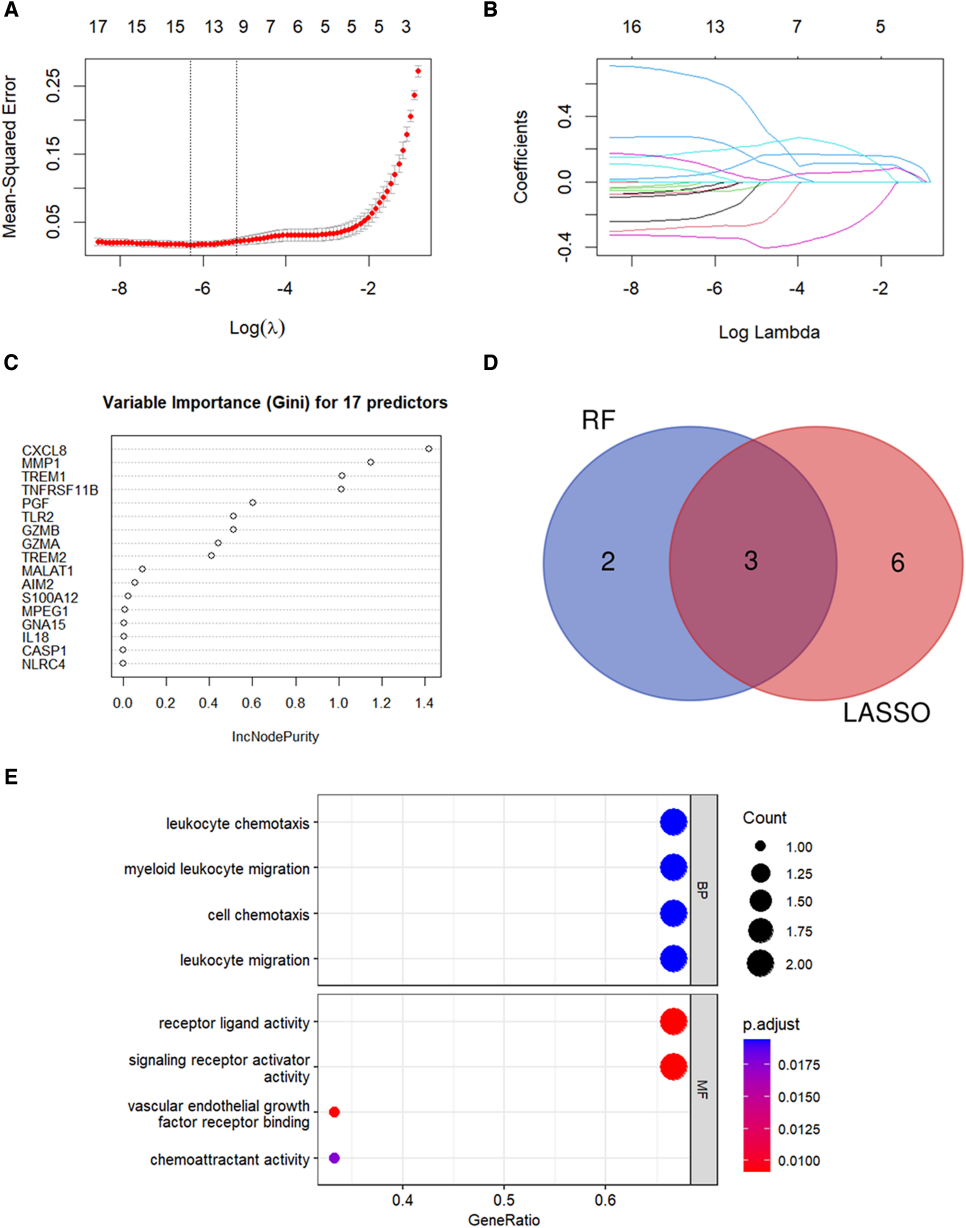
Identification of candidate diagnostic genes. (**A**,**B**) LASSO regression analysis was performed to filter the 17 pyroptosis-related genes. The dotted vertical lines show the optimal value through lambda.min and lambda.1se. Nine genes and their coefficients were selected based on the lambda.1se for further analysis. (**C**) RF algorithm was used to screen the diagnostic biomarkers. Top five genes with the variable importance scores >0.6 were selected. (**D**) Three diagnostic genes were identified by integrating the results of the LASSO regression and the RF analysis results. (**E**) GO analysis results for candidate diagnostic genes.

LASSO regression analysis was performed and nine candidate diagnostic genes were identified for CAVD (Figures 5A,B). Besides, RF analysis results identified five candidate diagnostic genes with importance scores above 0.6 (Figure 5C). Subsequently, we overlapped candidate genes from the LASSO model and the RF algorithm and identified three common candidate diagnostic genes, TREM1, TNFRSF11B, and PGF (Figure 5D). Functional enrichment analysis was performed to demonstrate the functions of three candidate diagnostic genes. As shown in Figure 5E, three diagnostic genes were enriched in the leukocyte chemokine and leukocyte migration among the BP categories, and chemoattractant activity among the MF categories.

### 3.4 Diagnostic performance of the model and potential biomarkers in detecting CAVD

**Figure 6:**
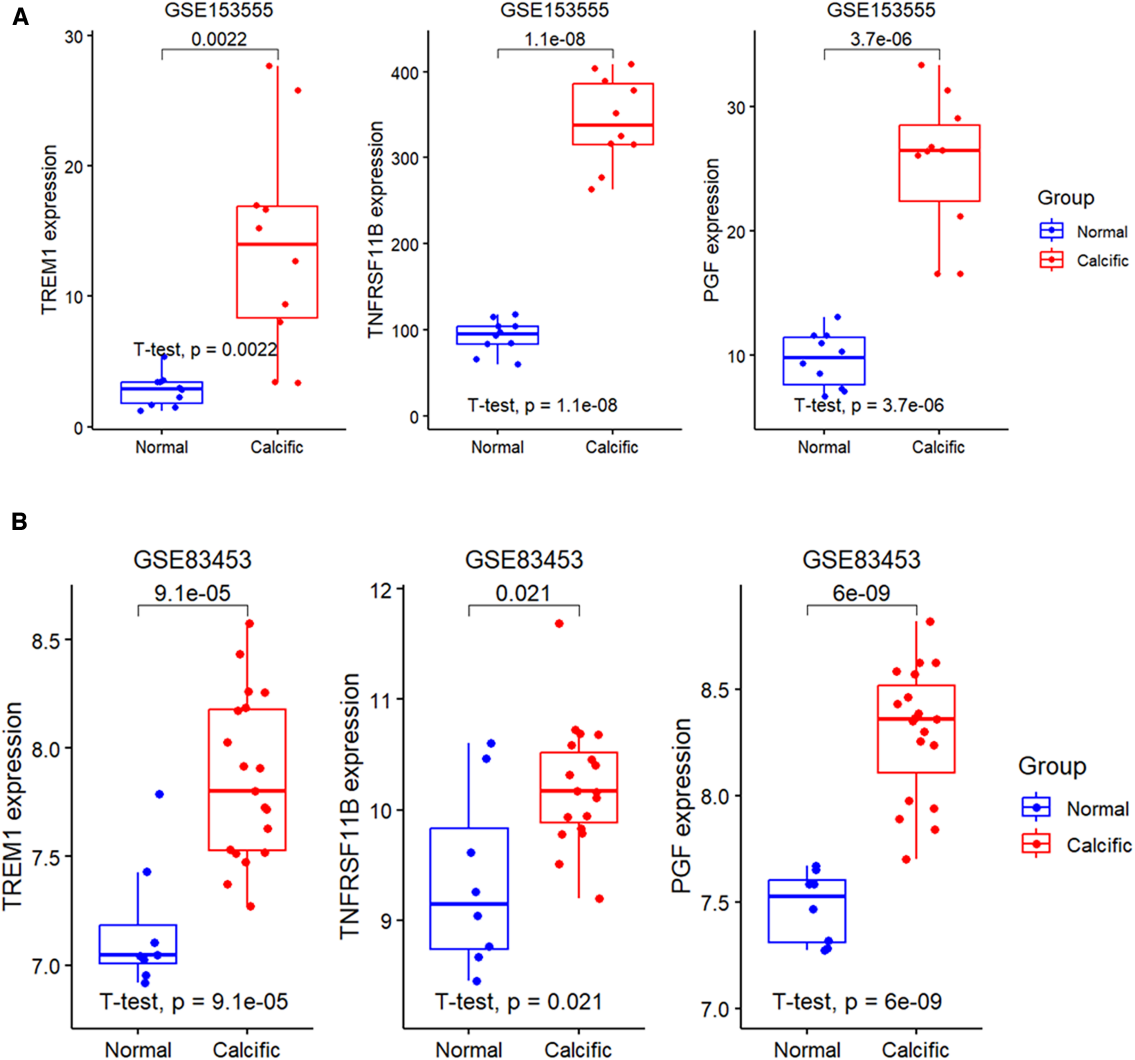
Expression levels of diagnostic genes in validation groups. (**A**) Validation of the expression profiles of the three diagnostic genes, namely, TREM1, TNFRSF11B, and PGF using the GSE153555 dataset. (**B**) Validation of the expression profiles of TREM1, TNFRSF11B, and PGF using the GSE83453 dataset.

The expression levels of the three candidate diagnostic genes, TREM1, TNFRSF11B, and PGF were significantly higher in the calcified group compared to the normal group in the GSE153555 and GSE83453 validation datasets (Figures 6A,B).

**Figure 7:**
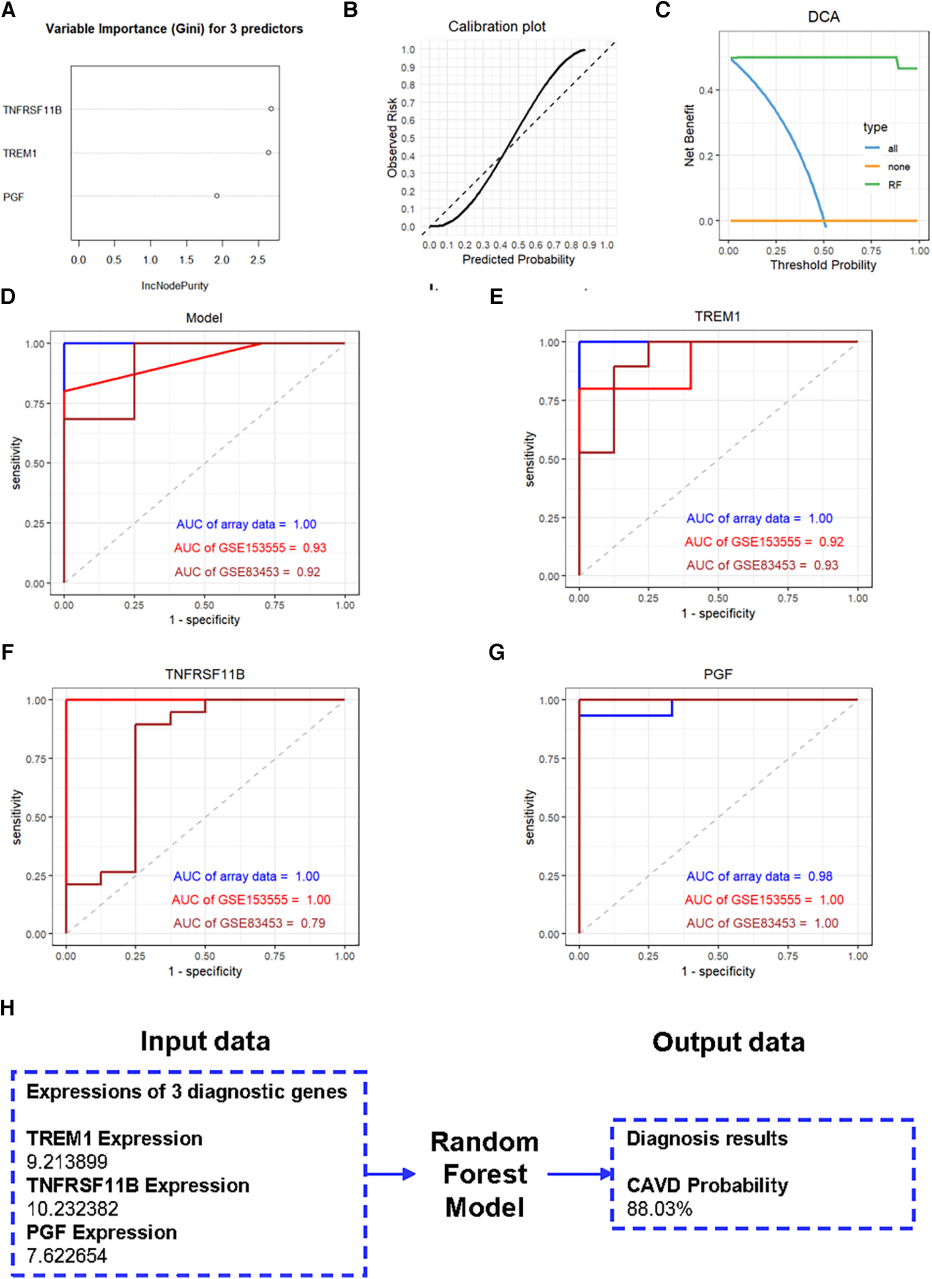
Construction of the diagnostic model. (**A**) Variable importance scores of TREM1, TNFRSF11B, and PGF in the diagnostic model. (**B**) The calibration plot of the model. (**C**) The DCA curve of the model. (**D-G**) ROC curve analysis results for the diagnostic model, TREM1, TNFRSF11B, and PGF in the training and the validation groups. (**H**) The website constructed to visualize the model for CAVD diagnosis.

We further constructed a diagnostic model based on these three genes using the RF method. The importance scores of TREM1, TNFRSF11B and PGF in the model are over 1.5 (Figure 7A). The calibration plot was applied to evaluate the accuracy of the model, and the results showed that actual and predicted probabilities were rather consistent (Figure 7B). The DCA curve showed that the RF model curve was highest, demonstrating patients could benefit from the diagnostic model (Figure 7C). ROC analysis was performed to determine the diagnostic value of the model and the three candidate genes. The diagnostic performance of the model was highly favorable for detecting CAVD, with an AUC value of 1.00 in the training set, an AUC value of 0.93 in the GSE153555 dataset and an AUC value of 0.92 in the GSE83453 dataset (Figure 7D). Furthermore, the three candidate genes also individually showed highly favorable performances for CAVD diagnosis. The AUC values for TREM1, TNFRSF11B, and PGF were 1.00, 1.00, and 0.98, respectively, in the training set, 0.92, 1.00, and 1.00, respectively, in the GSE153555 dataset and 0.93, 0.79 and 1.00, respectively, in the GSE83453 dataset (Figures 7E–G). A website was constructed to better visualize the model for CAVD diagnosis (https://cavddiagnosis.shinyapps.io/EDstr/) (Figure 7H). The input data is the expressions of TREM1, TNFRSF11B and PGF detected by array or high throughput sequencing. The output data is the probability of CAVD.

### 3.5 The associations between diagnostic genes, pyroptosis-related genes and CAVD-related genes

**Figure 8:**
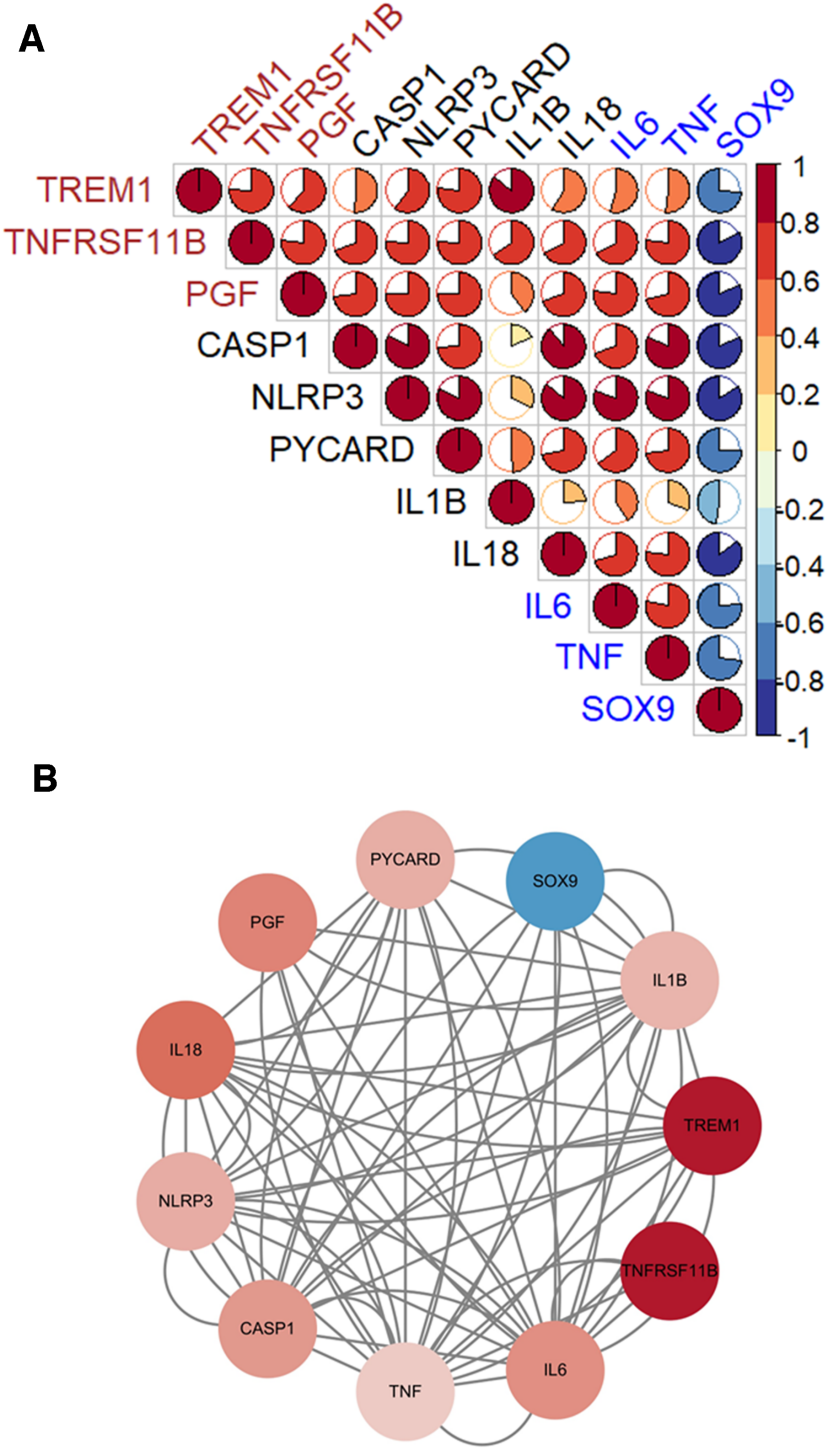
The associations between diagnostic genes, pyroptosis-related genes and CAVD-related genes. (**A**) Pearson's correlation analysis. Diagnostic genes and pyroptosis-related genes were positively correlated with IL6 and TNF, which promote the CAVD progression, but negatively correlated with SOX9 which suppresses the disease development. (**B**) The PPI networks. Complicated interactions between diagnostic genes (TREM1, TNFRSF11B and PGF), pyroptosis-related genes (CASP1, NLRP3, PYCARD, IL1B and IL18) and CAVD-related genes (IL6, TNF and SOX9).

Three diagnostic genes and 5 pyroptosis-related genes were positively correlated with two genes, IL6 and TNF, which promote the CAVD progression, but negatively correlated with SOX9 which suppresses the disease development (Figure 8A). The PPI analyses present intricate interactions between diagnostic genes (TREM1, TNFRSF11B and PGF), pyroptosis-related genes (CASP1, NLRP3, PYCARD, IL1B and IL18) and CAVD-related genes (IL6, TNF and SOX9) (Figure 8B).

### 3.6 Immune infiltration landscape of CAVD

**Figure 9:**
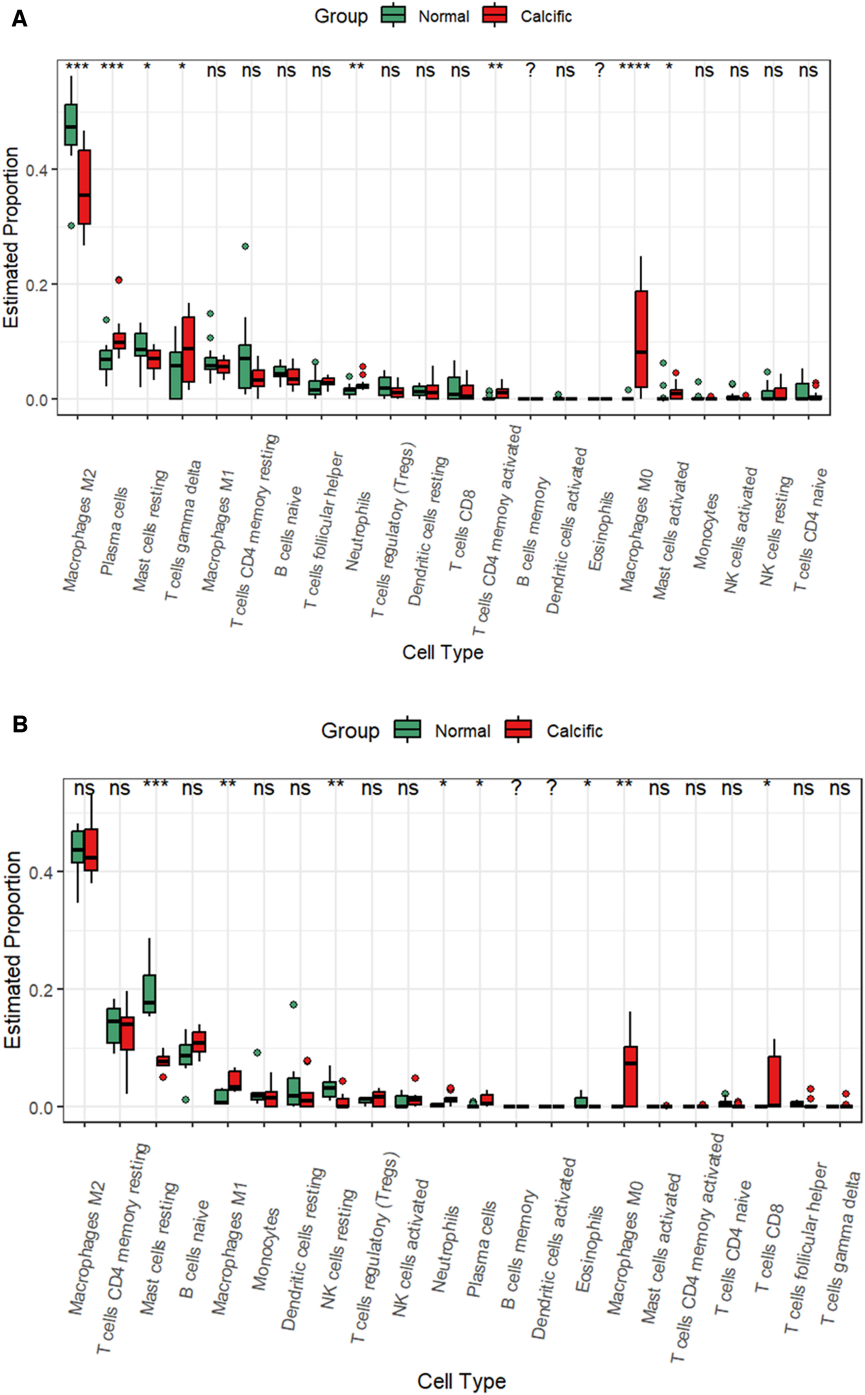
CIBERSORT analysis of the infiltrated immune cells in the valve tissues. (**A**) The differences in the proportions of different types of immune cells between the normal valve and calcified valve groups in the training cohort. (**B**) The differences in the proportions of different types of immune cells between the normal valve and calcified valve groups in the GSE153555 dataset. *p < 0.05.

CAVD and pyroptosis are closely associated with immune regulation (8, 16). Besides, functional enrichment analysis results suggested that the pyroptosis-related DEGs were mainly enriched in inflammatory responses. Therefore, we analyzed the status of the infiltration of immune cells by estimating the proportion of 22 different immune cell types to determine changes in the immune microenvironment between the calcified and the normal valves. In the training group, we observed significant differences in the proportions of eight immune cell types between the calcified and the normal valves. The calcified valves showed significantly higher proportions of M0 macrophages, plasma cells, neutrophils, activated CD4 memory T cells, gamma delta T cells, and activated mast cells, and significantly reduced proportions of M2 macrophages and resting mast cells compared to the normal valves (Figure 9A and Supplementary Figure S2). In the GSE153555 dataset, the proportions of proinflammatory cells, such as M1 microphages, neutrophils and plasma cells also were significantly higher in the calcified valves than in the normal valves. The proportions of resting mast cells and resting NK cells were significantly reduced in the calcified valves compared to the normal valves (Figure 9B). In the GSE83453 dataset, the proportion of resting NK cells was also lower in the calcified valves than in the normal valves (Supplementary Figure S3).

### 3.7 Correlation analysis of diagnostic genes with the infiltrated immune cell types in CAVD tissues

**Figure 10:**
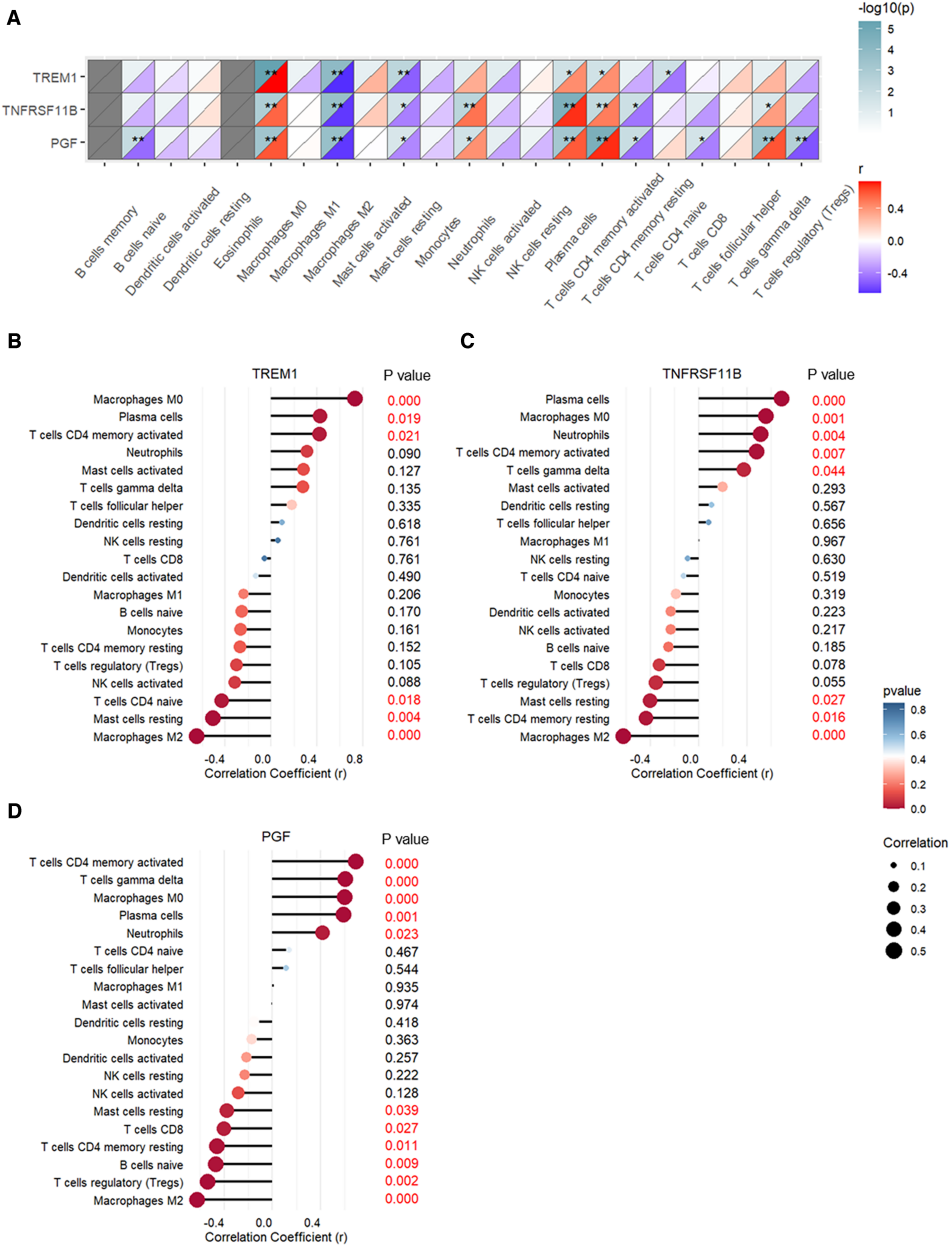
Pearson's correlation analysis between expression levels of TREM1, TNFRSF11B, and PGF and the proportions of different immune cell types. (**A**) Heatmap of the correlation analysis. (**B**) Bubble chart shows correlations between TREM1 expression levels and proportions of different immune cell types. (**C**) Bubble chart shows correlations between TNFRSF11B expression levels and proportions of different immune cell types. (**D**) Bubble chart shows correlations between PGF expression levels and proportions of different immune cell types.

The correlation between expression levels of the three CAVD diagnostic genes and the proportions of the immune cell types are shown in Figure 10. TREM1, TNFRSF11B, and PGF expression levels showed positive association with the proportions of M0 macrophages, plasma cells, and activated CD4 memory T cells, and negative association with the proportions of M2 macrophages and resting mast cells (Figure 10A). TREM1 was significantly associated with six types of immune cells and showed strongest correlation with the M0 macrophages (*r* = 0.73, *p* < 0.001) and M2 macrophages (*r* = −0.65, *p* < 0.001) (Figure 10B and Supplementary Figure S4). TNFRSF11B was significantly associated with eight immune cell types, and showed highest correlation with the plasma cells (*r* = 0.69, *p* < 0.001) and M2 macrophages (*r* = −0.62, *p* < 0.001) (Figure 10C and Supplementary Figure S4). PGF showed significant correlation with eleven immune cell types, and showed strongest correlation with the activated CD4 memory T cells (*r* = 0.69, *p* < 0.001) and M2 macrophages (*r* = −0.62, *p* < 0.001) (Figure 10D and Supplementary Figure S4).

### 3.8 Validation the expression differences of caspase-1

**Figure 11:**
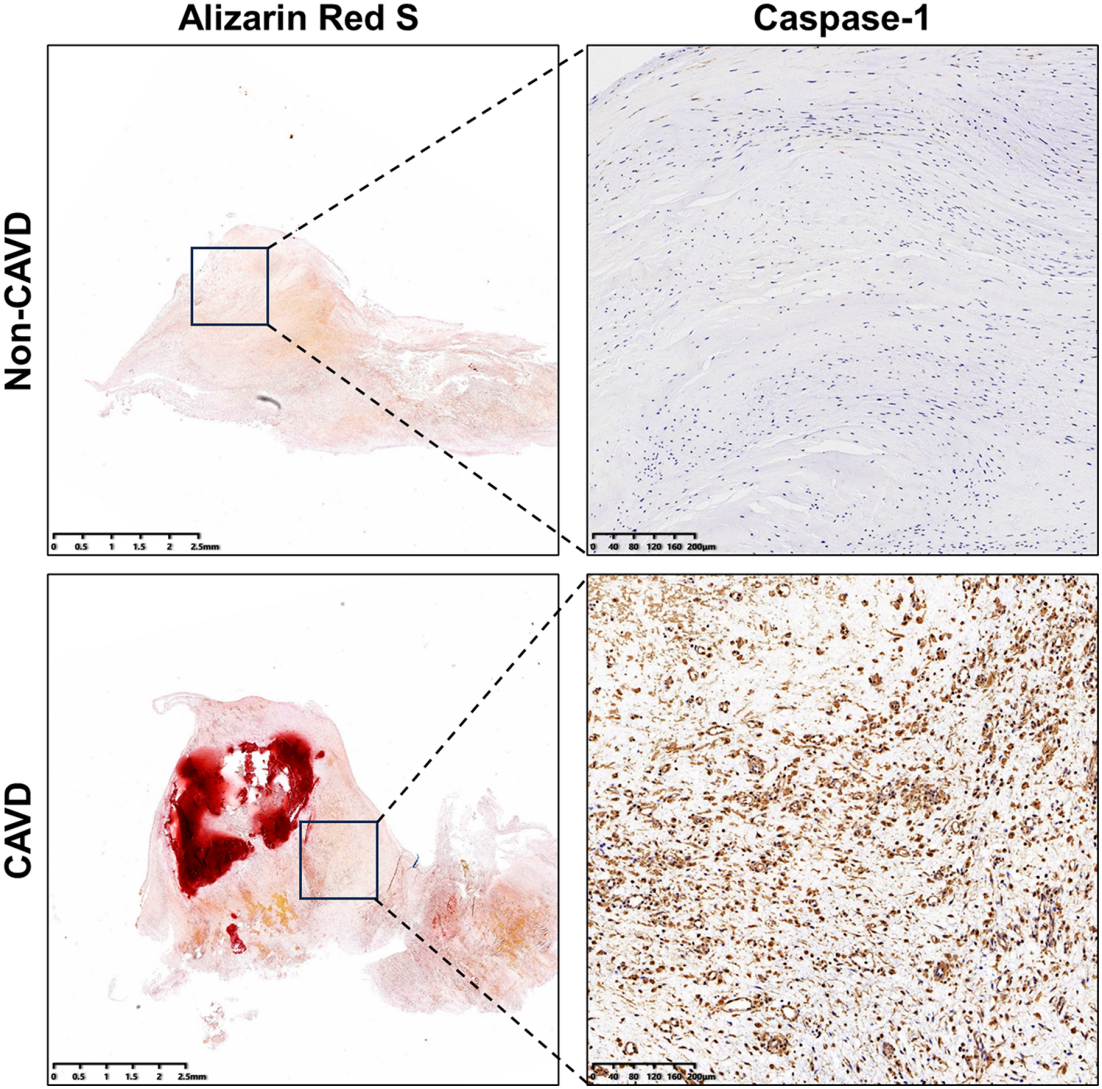
Levels of caspase-1 in calcific and non-calcific aortic valves. Immunohistochemistry showed higher expressions of caspase-1 in calcific valves than in non-calcific valves.

Immunohistochemistry was performed to further evaluate concentrations of the pyroptosis-related gene, caspase-1 in calcific and non-calcific aortic valves. The expression of caspase-1 was higher in calcific valves than in normal valves (Figure 11).

## 4 Discussion

CAVD affects approximately 9 million people around the world and is the second most common cause of cardiac surgery (21). Furthermore, the mechanism of CAVD is not fully understood. Therefore, it is necessary to investigate the underlying pathogenetic mechanisms and identify effective targets for the prevention and treatment of CAVD. Pyroptosis plays a significant role in several cardiovascular diseases, but its role in CAVD is not clear. Therefore, in this study, we investigated the role of pyroptosis on the development of CAVD and constructed a diagnostic model based on the differentially expressed pyroptosis-related genes. Furthermore, since both CAVD and pyroptosis are closely associated with inflammatory responses, we analyzed the correlation between diagnostic biomarkers identified in this study and the infiltrating immune cell types to determine the alterations in the immune microenvironment of CAVD.

We analyzed the gene expression data of calcified and normal valve samples and identified 805 DEGs. Subsequently, we analyzed the potential biological functions of the DEGs by functional enrichment analysis. GO analysis showed that CAVD-related DEGs were predominantly enriched in the pathways related to immune responses, including leukocyte migration and chemotaxis and chemokine activities. KEGG pathway analysis also demonstrated that CAVD was associated with the inflammatory pathways, including cytokine-cytokine receptor interactions and chemokine signaling pathways. Previous studies have demonstrated that inflammation plays a significant role in the occurrence and development of CAVD (16). Our findings are therefore consistent with previous reports. We then identified 17 pyroptosis-related DEGs among these 805 DEGs. The functional enrichment analysis showed that these pyroptosis-related DEGs were enriched in positive regulation of inflammatory responses and pyroptosis pathways. This further suggested strong association between CAVD, inflammation, and pyroptosis (8). Furthermore, LASSO regression and RF analyses identified three candidate diagnostic genes, namely, triggering receptor expressed on myeloid cells-1 (TREM1), TNF receptor superfamily member 11b (TNFRSF11B), and placental growth factor (PGF). The diagnostic model and each of the 3 candidate diagnostic genes demonstrated good diagnostic value with AUC values >0.75 in both the training and the validation groups.

TREM1 is a transmembrane receptor expressed on the neutrophils and monocytes, and functions as an amplifier of the immune responses (22). TREM1 promoted inflammation in patients with chronic obstructive pulmonary disease by activating caspase-1-induced pyroptosis (23). TREM1 is also involved in various cardiovascular diseases. TREM1 expression showed positive correlation with the plaque size and macrophage infiltration during the development of atherosclerosis (24). Besides, TREM-1-mediated pyroptosis was discovered in ox-LDL-treated endothelial cells (ECs) and could be the underlying reason of ECs dysfunction during atherosclerosis development (25). TREM1 also played a crucial role in myocardial infarction (MI) by inducing excessive inflammatory responses and triggering cardiac dysfunction (22). TREM1 is a promising target for cardiovascular diseases because several TREM1 inhibitors attenuate the formation of atherosclerotic plaques and improve cardiac function after MI in the experimental models (26, 27). However, the role of TREM1 in CAVD has not been reported. Our study found that TREM1 was upregulated in the calcified valves, which is consistent with previous research (28). And TREM1 was positively correlated with the M0 macrophages. This suggested that TREM1 could promote CAVD by inducing inflammation. Since the pathological processes underlying both atherosclerosis and CAVD are highly similar, TREM1 is a promising target to prevent and/or treat CAVD.

TNFRSF11B, also known as osteoprotegerin (OPG), is a secretory protein that interacts with receptor activator of nuclear factor kappa-B ligand (RANKL) and TNF-related apoptosis-inducing ligand (TRAIL) (29, 30). TNFRSF11B was associated with increased expression of NLRP3, caspase 1 and GSDMD, and induced osteoclast pyroptosis to protect the bone tissues (29). TNFRSF11B plays a significant role in several cardiovascular diseases, including coronary artery syndromes and myocardial infarction (31). TNFRSF11B is a biomarker of atherosclerosis. TNFRSF11B was positively correlated with the expression of intercellular adhesion molecule 1 (ICAM1), and increased the infiltration of monocytes into subendothelial spaces to promote atherosclerosis (32, 33). A therapeutic antibody targeting TNFRSF11B attenuated pulmonary arterial hypertension by reducing the proliferation and migration of the pulmonary artery smooth muscle cells (34). TNFRSF11B is also a biomarker for adverse outcomes in CAVD (35). TNFRSF11B levels increased in CAVD patients with NOTCH1 mutations (36). Consistent with previous studies (37), our results demonstrated overexpression of TNFRSF11B in the calcified valves compared with the normal valves. However, the role of TNFRSF11B in CAVD still needs further investigation.

Placental growth factor (PGF or PlGF) is a member of the vascular endothelial growth factor family (38). PGF is a direct target of miR-124-3p and promoted preeclampsia by inducing pyroptosis of the trophoblast cells (39). PGF also promoted infiltration of macrophages into the early atherosclerotic lesions and was strongly associated with the long-term prediction of coronary heart disease (40, 41). Treatment with anti- PGF antibody (alphaPlGF mAb) significantly reduced the size of early plaques and inhibited atherosclerosis progression in the ApoE-deficient mice (42). However, the functional role of PGF in CAVD has not yet been reported. Our study showed that PGF was upregulated in the calcified valves and was a promising diagnostic biomarker for CAVD. Furthermore, the expression levels of PGF showed positive correlation with the proportion of M0 macrophages in the valves. Therefore, based on the role of PGF in atherosclerosis, we speculate that PGF also contributes to the development of CAVD.

To further explore the role of diagnostic genes in pyroptosis and CAVD pathogenesis, we performed the correlation analyses and the PPI network of 3 diagnostic genes, 5 pyroptosis-related genes and 3 CAVD-related genes. TREM1, TNFRSF11B and PGF were positively correlated with caspase-1-induced pyroptosis genes (CASP1, NLRP3, PYCARD, IL1B and IL18). Previous studies also reported that these three diagnostic genes increased the expression of NLRP3 and promoted several diseases, such as septic cardiomyopathy via pyroptosis (29, 39, 43). Five pyroptosis-related genes were positively correlated with IL6 and TNF, but negatively correlated with SOX9. IL6 and TNF, two pro-inflammatory cytokines, contributed to the CAVD progression by initiating the immune cascade (16). SOX9, on the contrary, played a crucial role in preventing calcification (44). Pyroptosis was reported to involve in several cardiovascular diseases. Pyroptosis signaling pathway was activated in the epicardial adipose tissue (EAT), promoting the development of heart failure with preserved ejection fraction (HFpEF) (45). Suppressing NLRP3-mediated pyroptosis attenuated further myocardial damage in myocarditis (46). Caspase-1 inhibitor, VX-765, played an inhibitory role in SiNPs-induced pyroptosis and the development of cardiac hypertrophy (47). Pyroptosis-related genes (NLRP3, PYCARD, Caspase-1, IL-1β and Cleaved IL-18) were discovered to be upregulated in calcific valves, and a suppressor of NLRP3 could alleviate pyroptosis and CAVD progression (15). Thus, our results were consistent with the previous research. IL-1β and IL-18, two proinflammatory cytokines secreted during the pyroptosis process, were also promoted CAVD progression. IL-1β, through the NF-kB axis, promoted the release of inflammatory cytokines, such as IL6 (48). Deficiency of IL-1 receptor antagonist accelerated aortic valve inflammation and calcification (16). IL-18 participated in CAVD development via the NF-kB pathway (49). Therefore, the interactions between diagnostic genes, pyroptosis-related genes and CAVD-related genes play a vital role in the disease progression together and can be novel targets for treatment.

Immune responses and inflammation are closely associated with CAVD (16). Our data showed that the proportions of M0 macrophages and neutrophils were significantly higher in the calcified valves compared to the normal valves. M0 macrophages were strongly associated with all the three diagnostic genes, and neutrophils were significantly associated with the expression levels of TNFRSF11B and PGF. This demonstrated that the CAVD microenvironment was pro-inflammatory. During the pathological process of CAVD, monocytes infiltrate the valves via adhesion molecules and differentiate into M0 macrophages. Subsequently, M0 macrophages differentiate into M1 macrophages (pro-inflammatory) and M2 macrophages (anti-inflammatory) (16). Macrophage pyroptosis is also observed during atherosclerosis (50). Besides, macrophages interact with the vascular smooth muscle cells (VSMCs) and promote their phenotypic switching into macrophage-like cells; subsequent activation of the NLRP3 inflammasome in the VSMCs induced pyroptosis (51). Quercetin attenuated atherosclerosis by suppressing macrophage pyroptosis (50). However, the status and consequences of macrophage pyroptosis in CAVD remains to be characterized. Neutrophils produced cytokines and promoted the recruitment of macrophages into the valves (16). Neutrophil pyroptosis has been reported in several inflammatory diseases (52, 53). The functional role of neutrophils in CAVD pathogenesis remains to be explained. These findings demonstrated that inflammation played a key role in CAVD and the three CAVD diagnostic biomarkers identified in this study were associated with increased infiltration of immune cells and promotes CAVD development.

The diagnostic model was built based on the RF method. The RF method was applied in several diseases in previous studies (54, 55). Other machine learning methods, such as LASSO and SVM algorithm were also used to construct diagnosis or prognosis models (56). The translation of clinical applications is vital. Forest plots were applied to demonstrated the significance of biomarkers for prognosis prediction in hepatocellular carcinoma and head and neck squamous cell carcinoma (57, 58). A web-based calculator was established to visualize the prognostic model and predict the survival of patients with lung adenocarcinoma (59). Based on previous studies, we constructed an easy-to-use website (https://cavddiagnosis.shinyapps.io/EDstr/) for CAVD diagnosis. More datasets were included in our study compared to previous research on CAVD diagnosis models (14, 60). Besides, the AUC values of ROC in the training group and two validation groups were all over 0.9. And the calibration plot further validated the accuracy of our model, demonstrating it is suitable for disease diagnosis.

As far as we know, this is the first study to construct a diagnostic model based on the pyroptosis-related genes for CAVD. Furthermore, our study demonstrated positive correlation between the three diagnostic genes and several types of pro-inflammatory immune cells. This study has several limitations. Firstly, we did not assess the clinical characteristics in this study. We also did not include the clinical characteristics in our diagnostic model because it was difficult to obtain the information. Secondly, we identified the link between pyroptosis-associated DEGs and CAVD but the mechanism by which pyroptosis regulates CAVD needs to be further investigated and validated through biological experiments.

## 5 Conclusions

In summary, we performed comprehensive bioinformatics analysis and identified three pyroptosis-related diagnostic biomarker genes (TREM1, TNFRSF11B, and PGF) that were significantly associated with CAVD progression. Our findings provide new insights for investigating the functional role of pyroptosis in the development of CAVD in future studies.

## Data Availability

The datasets presented in this study can be found in online repositories. The names of the repository/repositories and accession number(s) can be found below: Gene Expression Omnibus, GSE51472, GSE12644 and GSE153555.
