# Supplementary material for: Identification of pyroptosis-associated genes with diagnostic value in calcific aortic valve disease
Source: Front Cardiovasc Med. 2024 Jan 25;11:1340199. doi: 10.3389/fcvm.2024.1340199 (PMC10850341; doi:10.3389/fcvm.2024.1340199)

Supplementary Material

**Supplementary Figure 1.** Gene expression analysis results of the combined GSE51472 and GSE12644 datasets before and after batch correction.


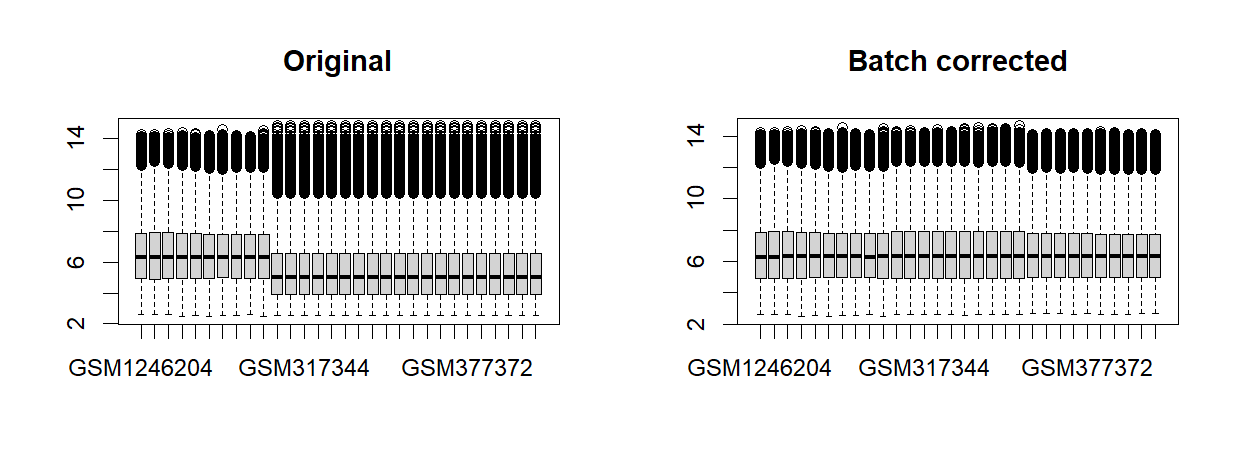


**Supplementary Figure 2.** CIBERSORT analysis. The proportions of 22 types of immune cells in normal and calcific aortic valves of the training group.


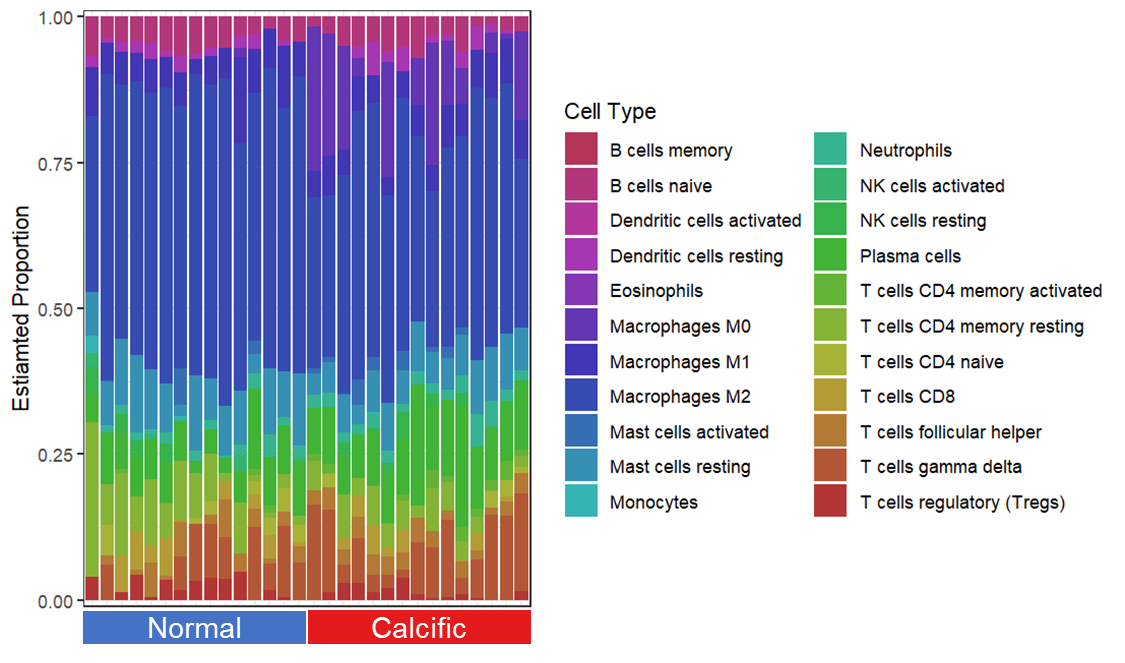


**Supplementary Figure 3.** CIBERSORT analysis of the infiltrated immune cells in the valve tissues. The differences in the proportions of different types of immune cells between the normal valve and calcified valve groups in the GSE83453 dataset. * p <0.05.


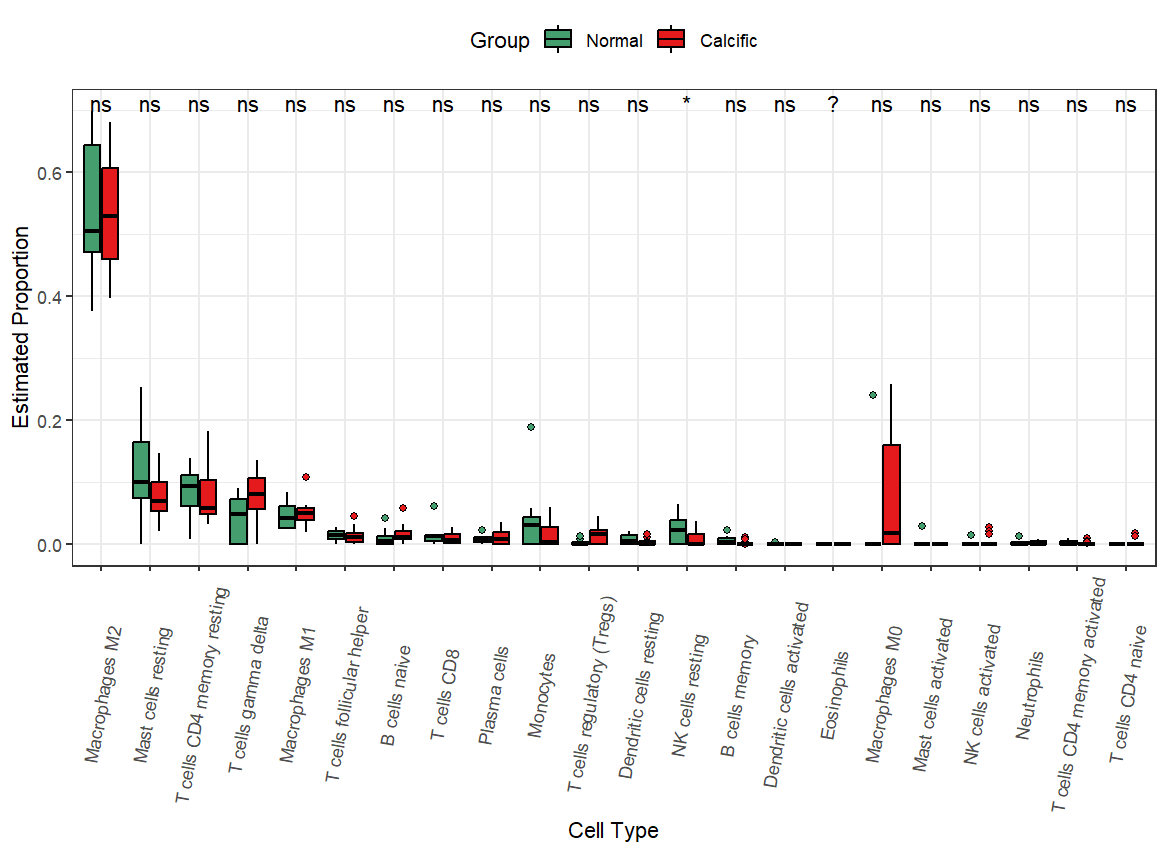


**Supplementary Figure 4.** Correlation analysis of diagnostic genes and five types of immune cells (M0 macrophages, M2 macrophages, resting mast cells, plasma cells, activated CD4 memory T cells).


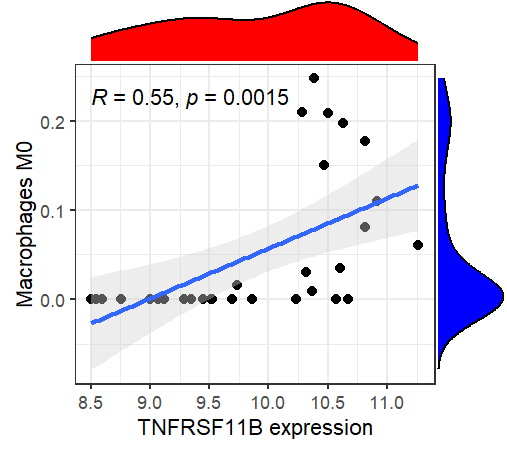

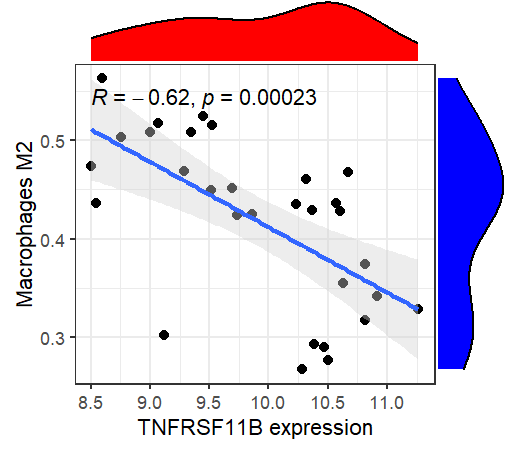

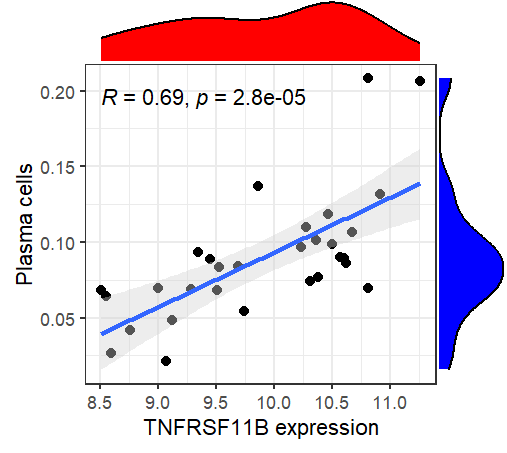

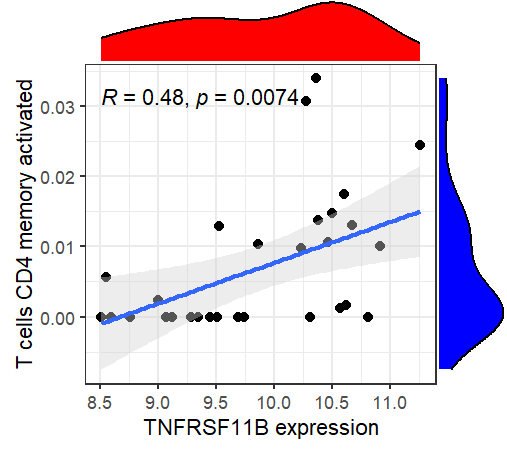

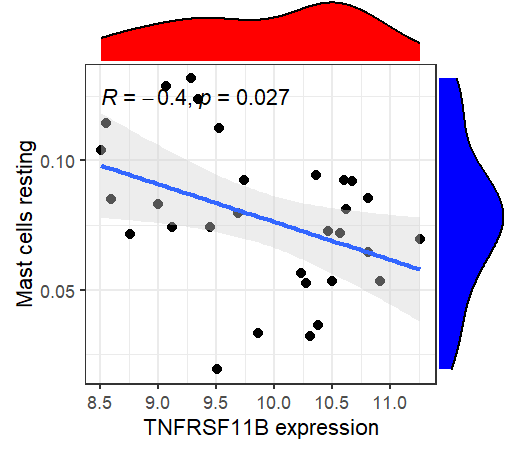

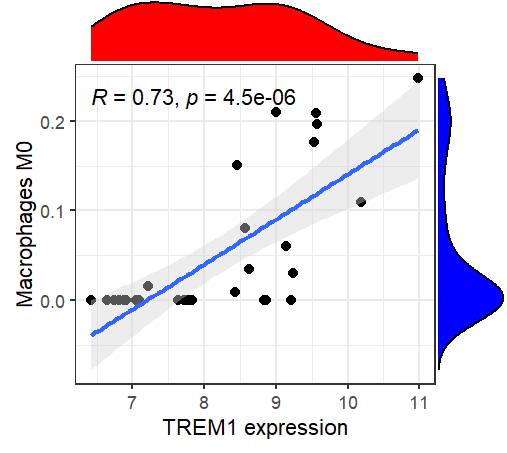

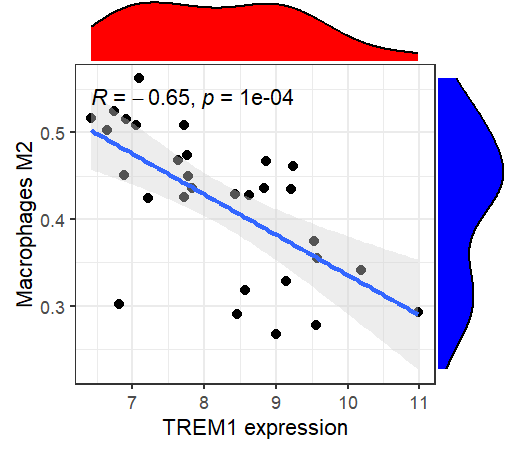

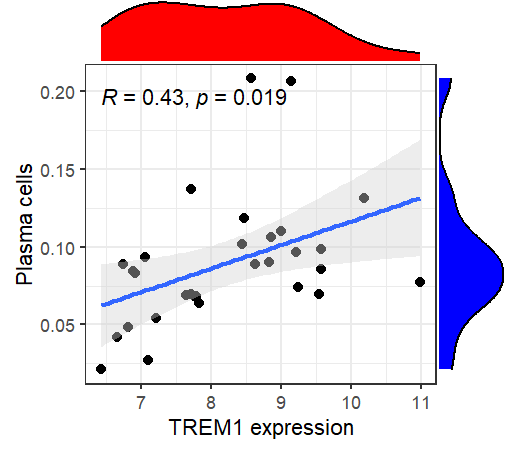

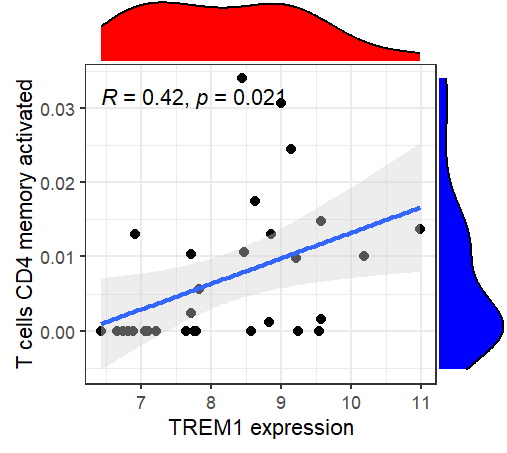

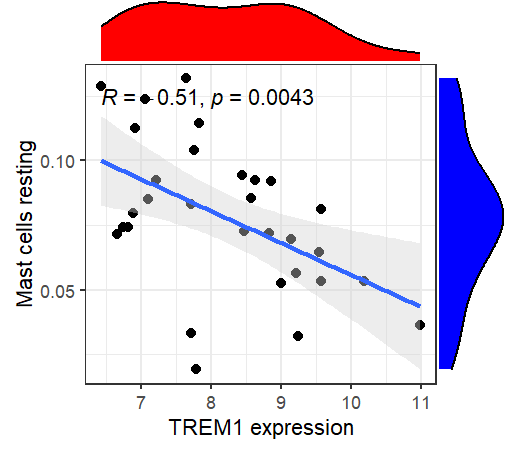

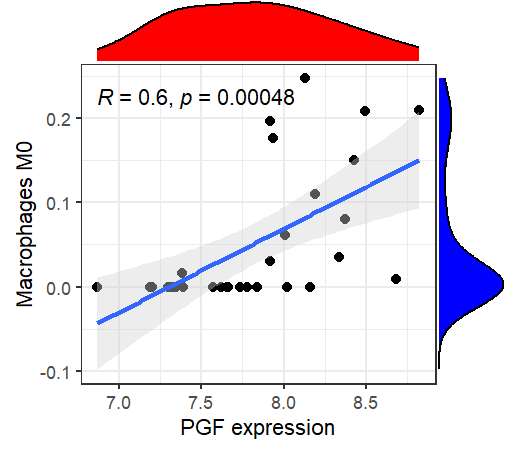

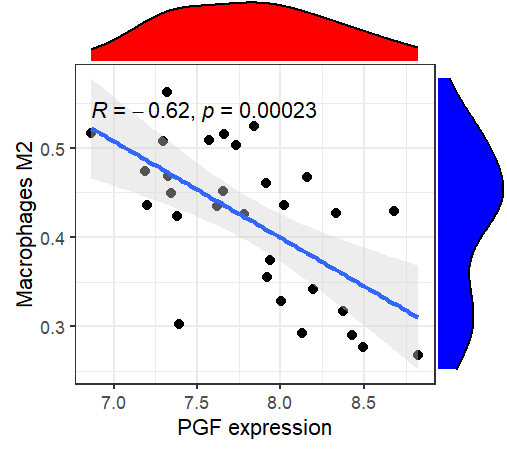

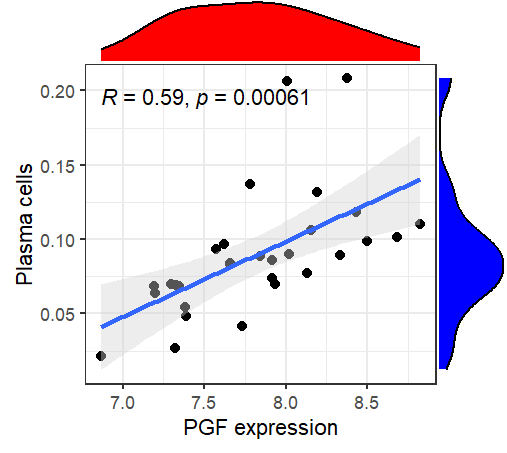

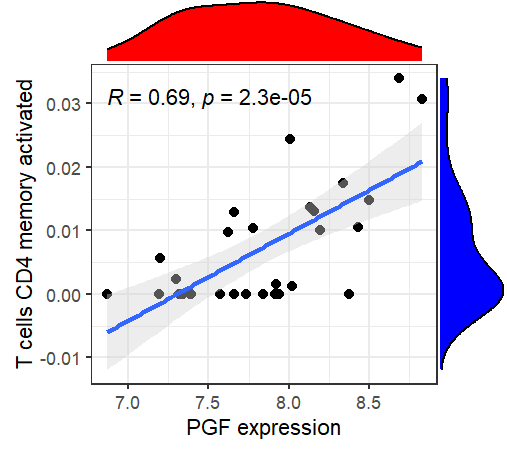

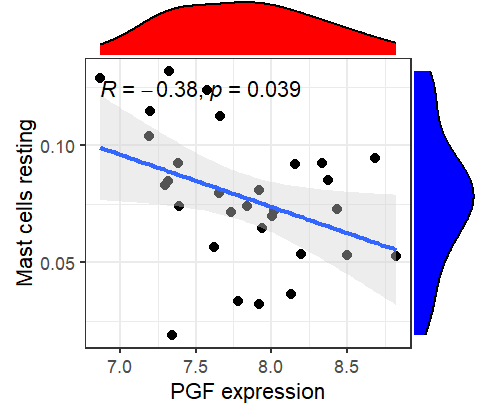

Supplement: Supplementary file 1 [file Table1.docx]
